# Research Progress on the Effect of Epilepsy and Antiseizure Medications on PCOS Through HPO Axis

**DOI:** 10.3389/fendo.2021.787854

**Published:** 2021-12-21

**Authors:** Shuang Li, Linhai Zhang, Nian Wei, Zhenzhen Tai, Changyin Yu, Zucai Xu

**Affiliations:** Department of Neurology, Affiliated Hospital of Zunyi Medical University, Zunyi, China

**Keywords:** epilepsy, sodium valproate, polycystic ovary syndrome, female, hypothalamic-pituitary-ovarian axis

## Abstract

Epilepsy is a common chronic neurological disease that manifests as recurrent seizures. The incidence and prevalence of epilepsy in women are slightly lower than those in men. Polycystic ovary syndrome (PCOS), a reproductive endocrine system disease, is a complication that women with epilepsy are susceptible to, and its total prevalence is 8%–13% in the female population and sometimes as high as 26% in female epilepsy patients. The rate of PCOS increased markedly in female patients who chose valproate (VPA), to 1.95 times higher than that of other drugs. In addition, patients receiving other anti-seizure medications (ASMs), such as lamotrigine (LTG), oxcarbazepine (OXC), and carbamazepine (CBZ), also have reproductive endocrine abnormalities. Some scholars believe that the increase in incidence is related not only to epilepsy itself but also to ASMs. Epileptiform discharges can affect the activity of the pulse generator and then interfere with the reproductive endocrine system by breaking the balance of the hypothalamic–pituitary–ovarian (HPO) axis. ASMs may also cause PCOS-like disorders of the reproductive endocrine system through the HPO axis. Moreover, other factors such as hormone metabolism and related signalling pathways also play a role in it.

## 1 Introduction

In recent years, the incidence of epilepsy in China has been on the rise. According to epidemiological surveys, epilepsy affects 70 million people worldwide (1, 2) and approximately 10 million people in China (3); the incidence in females is slightly lower than that in males (4), and 1.3 million females with epilepsy in the United States are in the fertile stage (2). Epilepsy requires long-term treatment, mainly oral anti-epileptic drugs, but their chronic use will produce certain adverse effects on some organ systems (5). In recent years, disorders of the reproductive endocrine metabolism system, such as polycystic ovary syndrome (PCOS), have attracted extensive attention from researchers (6). The prevalence of PCOS varies in different studies due to the different diagnostic criteria and to ethnic differences, ranging from 8% to 13% in the general population (7), but the incidence in patients with epilepsy is approximately 3.1%–20% (8) and is sometimes as high as 26% (9). PCOS is found in approximately 10%–25% of women with epilepsy (WWE) (10) and causes infertility in those who are at the reproductive stage (11). The main clinical features include hyperandrogenemia (HA), chronic anovulation, polycystic ovary (PCO), insulin resistance (IR)/hyperinsulinemia, obesity, dyslipidemia, and other metabolic changes. Some scholars believe that the reason women suffer from both PCOS and epilepsy is related to epilepsy (12), and some scholars believe that it at least, in part, results from the effects of anti-seizure medications (ASMs), especially valproate (VPA) (13, 14); hence, most scholars believe that the emergence of PCOS in patients with epilepsy is not only related to epilepsy but also to ASMs (10, 15, 16), as they could affect reproductive health and secretion abnormalities through the hypothalamic–pituitary–ovarian (HPO) axis. The occurrence of epilepsy and the pharmacological action of the anti-epileptic drug VPA can target some substrates and affect hormone levels, causing disorders of the reproductive endocrine and metabolic systems, including the limbic system, liver, hypothalamus, pituitary, ovary, and adipose tissue (17). This article aimed to review the progress of research on how epilepsy, VPA, or other ASMs affect the development of PCOS in WWE through the HPO axis. At the same time, it also introduces other possible mechanisms that cause the occurrence of PCOS. It is hoped to be favorable for clinical neurologists and obstetricians to prevent and treat this disease.

## 2 The Core Mechanism of PCOS Is Related to the Abnormal HPO Axis

PCOS is a prominent reproductive endocrine disorder in women of childbearing age (7, 10, 18), affecting 6%–10% (19, 20), and is related to genetic factors, environmental factors, and some other causes, such as the use of ASMs, epilepsy, and obesity; it is thus a multifactorial disease (18, 21, 22). This syndrome can cause HA, PCO, ovulation disorders (ODs), elevated levels of luteinizing hormone (LH), and an imbalance in the ratio of luteinizing hormone to follicle stimulating hormone (LH/FSH); some patients also have metabolic changes such as IR/hyperinsulinemia, obesity, and dyslipidemia. Currently, the pathogenesis of PCOS is not yet completely understood and is still under continuous research, but a number of studies have suggested that HPO axis dysfunction, elevated androgen, IR/hyperinsulinemia, elevated LH/FSH ratio, obesity, oxidative stress, and impaired negative feedback regulation of steroid hormones can all promote the occurrence and development of PCOS (11, 21, 23–25). The core mechanism is the abnormal function of the HPO axis. The hypothalamus pulse generator regulates the pulse release of gonadotropin-releasing hormone (GnRH) neurons, which act on the pituitary gland and regulate the secretion of LH and FSH. The latter acts on the ovaries to stimulate follicular growth and produce estradiol so as to effectively coordinate the function of the HPO axis (26–29). The hormones released by the pituitary and ovary can also negatively regulate the secretion of hormones in the hypothalamus–pituitary system and maintain the homeostasis of the reproductive endocrine system. Since there are no receptors for gonadal hormone and gonadotropin in GnRH neuron cells, the negative feedback regulation mechanism of androgens and estradiol on the hypothalamus is attributed to neurotransmitters and neuropeptides (30–32). When some factors cause the release of GnRH, it will promote the secretion of pituitary hormones, including the increase of the level of LH and the increase of LH/FSH, which in turn increase androgen secretion in the ovary. Androgens can affect the negative feedback regulation of estrogen, causing polycystic changes in the ovary and ODs. Finally, it leads to PCOS (25, 33). The cause of PCOS is not clear, and the clinical manifestations are also diverse. At present, the disease cannot be cured, and symptomatic treatment is the main focus. Adjustments in lifestyle, as the first-line treatment, include dieting, exercise, or weight loss, followed by drug treatment, which needs to be individualized. Clomiphene and letrozole can be chosen to induce ovulation, metformin to improve IR, and oral contraceptives and spironolactone to reduce androgen levels (11, 18, 21, 34, 35). In addition, there are also studies suggesting that sex hormone binding globulins (SHBGs) can serve as indicators and therapeutic targets for hyperandrogenism in patients with PCOS (36, 37).

## 3 Related Research Works on Epilepsy and PCOS

Epilepsy is a chronic and recurrent disease caused by the highly synchronized abnormal discharge of brain neurons, especially those closely related to the limbic system, such as the hippocampus and amygdala. Reproductive endocrine abnormalities are common in female patients with epilepsy, including hyperandrogenemia, ODs, PCO morphology, PCOS, and menstrual disorders (10, 12, 16). The brain mainly regulates and controls the HPO system and affects the release of hormones at all levels of the hypothalamus–pituitary–gonad axis through nerves and the neuroendocrine system (22). That a certain connection may exist between epilepsy and PCOS was first proposed in 1984 (12). Recently, in a clinical study, WWE were more inclined (52.3%) to develop PCOS than women without epilepsy (18.3%) (10). In addition, a few other studies pertaining to reproductive endocrine disorders in patients with epilepsy have also confirmed that epilepsy could increase the hazards of reproductive endocrine disorders in WWE (16, 38, 39).

In the hypothalamus, there is a key structure, namely, the GnRH pulse generator, that can affect the activity of GnRH neurons, regulate the pulsatile release of GnRH, and affect the reproductive endocrine system through the HPO axis (22). In the hypothalamus, the area producing, secreting, and regulating GnRH receives a wide range of straight connections from the cerebral hemispheres, in particular the temporolimbic structure, the most prominent of which is the amygdala (38, 40–42). The amygdala is generally divided into two areas whose functions are different in some aspects, namely, the medial cortical nucleus group and the basolateral nucleus group. The medial cortical nucleus group stimulates the release of GnRH in the hypothalamus, while the basolateral nucleus group inhibits the release of GnRH in the hypothalamus (40, 42). Therefore, due to the close anatomical relationship and the extensive and direct fiber connection between the limbic system and the hypothalamus, the epileptiform discharges will act on some hypothalamic structures that produce, secrete, and regulate GnRH, such as the arcuate nucleus and paraventricular nucleus, to increase the frequency or amplitude of the GnRH pulse (10, 43), which in turn enhances the LH pulse release and increases the LH/FSH ratio (44). This causes abnormalities in the hormone secretion levels in the HPO axis, eventually resulting in the occurrence of PCOS; consequently, reproductive endocrine dyscrasia in patients with epilepsy could be reasonably expected (38).

The contents of GnRH in the hypothalamus on both sides are different, and it is much more abundant on the right side than on the left side (45). The laterality of epilepsy is an important factor affecting reproductive endocrine disorders in WWE (38, 46). A close connection exists between left temporal lobe epilepsy (TLE) and higher pulse frequency GnRH secretion, which in turn is associated with higher LH/FSH ratio and serum testosterone levels (38). In addition, a few researchers believe that the left TLE is closely connected with the occurrence of PCOS (38, 43), while a close connection exists between right TLE and lower GnRH pulse frequency, which could reduce the levels of LH and estradiol, which are characteristic of HA (46). A recent animal study has discovered that in a TLE model established with the injection of kainic acid (KA), all mice injected with KA had increased excitability of GnRH neurons (47). So far, research works on the effects of epilepsy on GnRH neurons have been limited to animal models, and data on humans are not available. We still do not know whether epilepsy directly affects the function of GnRH neurons or indirectly through other mediators. In addition, as seizure patterns are known to change with the reproductive cycle, it is not clear whether the effects of epilepsy on GnRH neurons change with women’s reproductive cycles.

**Figure 1:**
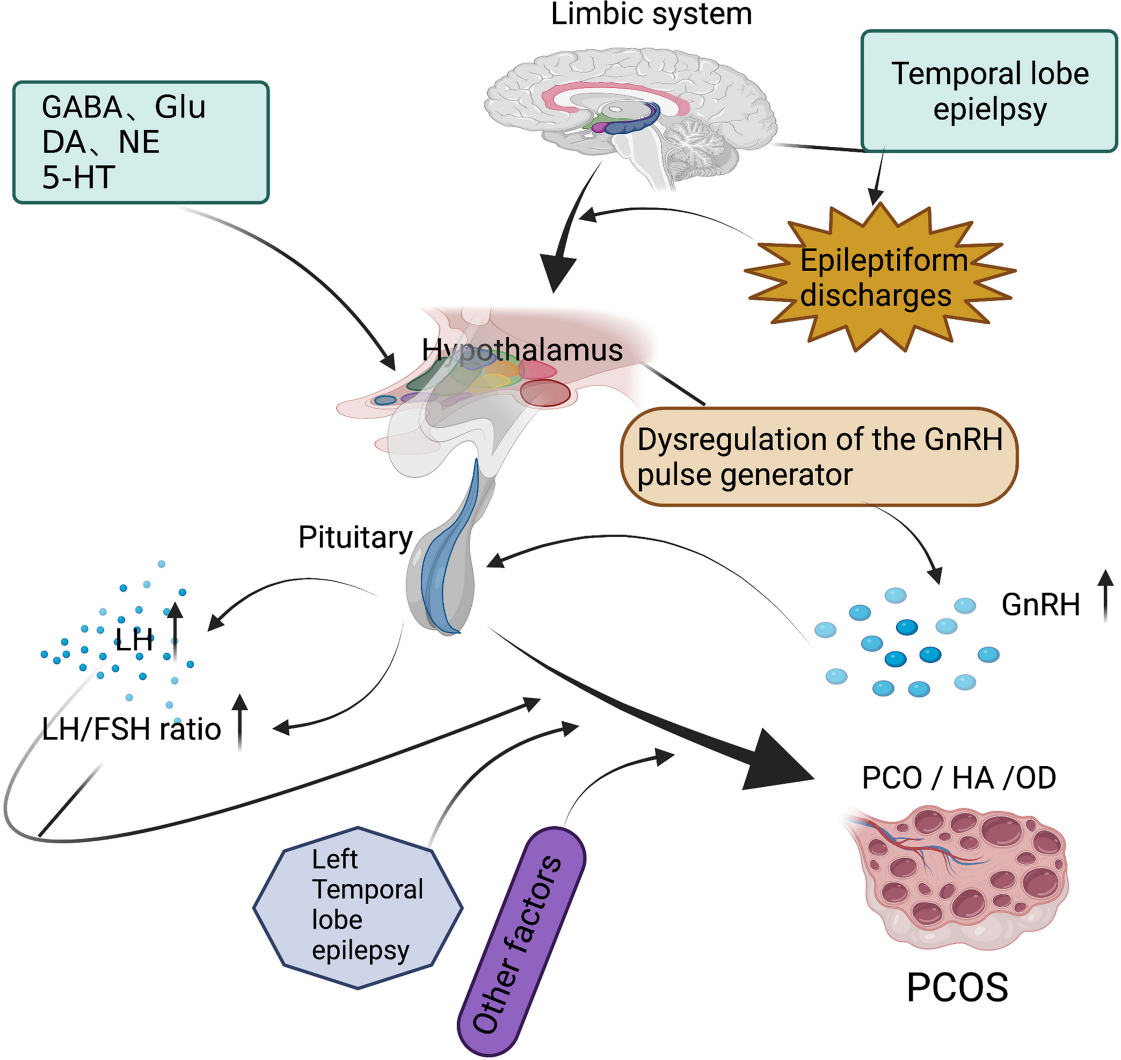
Regarding the mechanism of polycystic ovary syndrome (PCOS) in temporal lobe epilepsy (TLE), epileptiform discharges toward the hypothalamus interfere with the activity of the gonadotropin-releasing hormone (GnRH) pulse generator and neurotransmitters affect the excitability of GnRH neurons to enhance the frequency of GnRH secretion, which in turn leads to an increase in luteinizing hormone (LH) secretion and an increase in the ratio of LH/follicle stimulating hormone (FSH). Laterality (more common in left TLE) and other factors, such as age at seizure onset, type of epilepsy, and activity of epilepsy, are all involved in PCOS.

The incidence of PCOS was associated with age at onset of seizures (younger than 16 years), but not with seizure type or seizure frequency (16). However, it has been suggested that PCOS is more common in focal epilepsy, especially TLE (12, 38). Some studies have shown that abnormalities such as HA or PCOS are more likely to occur in patients with idiopathic generalized epilepsy than in patients with site-related epilepsy (15). The inconsistent results of these studies may be related to the following factors: 1) the sample size of the study; 2) differences in the clinical characteristics of the patients, such as age of the patients, age of onset, and other factors; and 3) the different diagnostic criteria for HA/PCOS. But, in general, patients with TLE are indeed a high-risk group of PCOS. On the one hand, since TLE is a common type of epilepsy in women of childbearing age, a large number of PCOS studies have focused on patients with TLE. On the other hand, it is attributed to the close anatomical connection and the extensive and direct fiber connection between the temporolimbic structure and the hypothalamus (**Figure 1**).

As is known, the epileptic seizures in patients are often accompanied by the change of neurotransmitter level. The imbalance between the excitatory neurotransmitter and inhibitory neurotransmitter leads to an abnormal neuron discharge, which is an important pathogenesis of epilepsy, mainly including γ-aminobutyric acid (GABA), dopamine (DA), norepinephrine (NE), and 5-hydroxytryptamine (5-HT). These neurotransmitters inhibit seizures; when a seizure occurs, their levels will decrease in the central nervous system. On the other hand, glutamate (Glu) and acetylcholine induce seizures; when a seizure occurs, their levels will increase in the central nervous system (48). At the same time, neurotransmitter changes can regulate the excitability of GnRH neurons (49, 50). GnRH neurons in the hypothalamus are the ultimate common pathway of the central reproductive regulation system, and their migration, changes in synaptic plasticity, and secretion are precisely regulated by many signaling molecules, among which is GABA (51). Some people believe that when GABA binds to the A receptor, it depolarizes GnRH neurons and stimulates the secretion of GnRH. Others believe that it will hyperpolarize GnRH neurons and inhibit GnRH secretion. In general, it mainly promotes the secretion of GnRH (52–54). Differences in the results may be related to the physiological stage of the body and the delay of GABA receptor signal transition. The exact mechanism is not yet clear (55). GABA and Glu participate in the negative feedback regulation of sex hormones (30, 32). When their levels are abnormal, they can alter the synaptic inputs or discharge rates of GnRH neurons and then promote GnRH neurons to release GnRH (56, 57). Glu may regulate the excitability of GnRH neurons and promote the secretion of GnRH when it binds to corresponding receptors. The receptors for Glu include ionotropic receptors [*N*-methyl-d-aspartate receptor (NMDAR), AMPA receptor (AMPAR), and kainic acid receptor (KAR)] and metabotropic receptors (mGluRs) (58). These receptors seem to be expressed in GnRH neurons. Dopamine can inhibit the excitability of GnRH neurons by acting on D1 and D2 receptors or affecting GABA/Glu postsynaptic currents (49, 59), which is consistent with previous research findings. Epileptiform discharge may also make women susceptible to PCOS through the depletion of dopamine in the brain. Dopamine can reduce the secretion of LH from the pituitary gland and can also act on the median bulge to inhibit GnRH secretion (12). The effect of 5-hydroxytryptamine (5-HT) on GnRH neurons is biphasic. The activation of the 5-HT2A receptor increases GnRH neuronal activity through the PKC (protein kinase C) pathway and promotes the release of GnRH, while the activation of the 5-HT1A receptor causes GnRH neurons to be hyperpolarized and inhibits GnRH secretion through adenylate cyclase (60). NE acts on the A1 and B receptors of GnRH neurons, mainly inhibiting the hormone release of GnRH neurons (61). Therefore, based on the relationship between neurotransmitters and GnRH neurons, the neurotransmitter in patients with epilepsy may cause reproductive endocrine dysfunction through the HPO axis at the hypothalamic level.

Besides, the level of prolactin (PRL) will increase in patients with epilepsy, so epilepsy could disrupt the hormone secretion balance of the HPO system by affecting the negative feedback of PRL (62). In short, epilepsy can affect the HPO axis through abnormal discharge, change the level of the central nervous system neurotransmitter, and change the level of PRL, leading to PCOS or other reproductive endocrine disorders in patients.

## 4 The Effect of ASMs on PCOS

ASMs have experienced so many years of development, and third-generation drugs have been on the market. However, the association between these drugs and PCOS is gradually increasing. In addition to VPA, the relevance between other ASMs and PCOS has attracted more and more attention. We will explain it from two aspects: VPA and other ASMs.

### 4.1 VPA Affects the Reproductive Endocrine System

VPA is a traditional anti-epileptic drug. It is mainly used to treat idiopathic generalized epilepsy and can also be used to treat focal epilepsy (63). Due to its teratogenicity, cognitive development impairment, and autism risks (63–65), its use in women of childbearing age is strictly regulated, but is sometimes inevitable (66, 67). Recently, a single-center cohort study reported that almost one-third of WWE were receiving VPA treatment, and most of them were of childbearing age (67). However, a meta-analysis showed that the incidence of PCOS in female patients who choose VPA was significantly increased, and its incidence was 1.95 times higher than that of other drugs (68). It also exerts an enormous function in controlling epilepsy, mainly through the following mechanisms: 1) enhancing the effect of the inhibitory neurotransmitter GABA as a GABA activator and 2) blocking the voltage-gated sodium channels and T-type calcium channels (69). The main substrates are the liver and the HPO axis, causing abnormal levels of sex hormones (70, 71). Several research works have indicated that the occurrence of reproductive endocrine disorders in patients taking VPA increased significantly (5, 10, 72–74), especially in patients who started using VPA while younger than 20 years, indicating that the reproductive endocrine function of young WWE is more likely to be affected by VPA (74, 75). However, some studies have found that VPA treatment has no serious impact on reproductive endocrine function and that it is safe to use (76). Therefore, there may be some controversies with respect to the adverse influence of VPA on the reproductive endocrine system, and a large number of samples are needed for extensive research. However, consensus has been basically reached on the adverse effects of VPA on the reproductive endocrine system. How VPA affects the occurrence and development of hyperandrogenemia, ODs, polycystic ovaries, IR, and weight gain is not yet fully understood; hence, research and exploration are still ongoing.

#### 4.1.1 Does VPA Affect PCOS Through the HPO Axis?

Because patients treated with VPA are prone to reproductive endocrine dysfunction, there must be a correlation between VPA and the HPO axis. Firstly, VPA can lead to an increase in GABA content in the brain, which stimulates the secretion of GnRH. Secondly, VPA, as a histone deacetylase inhibitor, may inhibit the transcription of the *GnRH1* gene in the hypothalamus and promote changes in the plasticity of GnRH neurons (77). This effect can also reverse the differentiation of LH and FSH cells into PRL cells, resulting in abnormal sex hormone levels and destruction of the hypothalamic–pituitary–gonadal (HPG) axis (78). Thirdly, the increased level of leptin and the decreased level of adiponectin in patients treated with VPA (79–82) and their receptors were expressed in the hypothalamus and pituitary (83). Leptin can indirectly regulate the excitability of GnRH neurons through kisspeptin neurons and other interneurons and promote the release of GnRH (84). Adiponectin inhibits GnRH secretion by activating the AMP kinase pathway (83). Consequently, VPA can also cause reproductive and endocrine abnormalities through leptin and adiponectin. Finally, patients treated with VPA have elevated insulin levels, which regulate GnRH secretion at the hypothalamus level while binding to the corresponding receptor (85). Of course, abnormal sex hormone levels such as androgen and estrogen will also affect the HPO axis. Animal studies have confirmed that VPA may affect the differentiation of GnRH neurons and the activation of GnRH pulse generators by increasing the concentration of GABA in the central nervous system (86, 87). Therefore, in conclusion, VPA may disrupt the balance of the HPO axis by regulating GnRH secretion at the hypothalamic level through a variety of mechanisms, leading to the occurrence of PCOS or other reproductive endocrine abnormalities in women.

#### 4.1.2 Other Possible Mechanisms of HA Caused by VPA

The most basic and main clinical feature of PCOS is elevated androgen levels, and approximately 80% of women with elevated androgen levels are diagnosed with PCOS (88), including biochemical or clinical androgen elevations. A serum level exceeding 4.2 nmol/L (10 mg/L) indicates hyperandrogenism (76). When androgen is elevated, the clinical manifestations are hirsutism and acne. The elevated androgens mainly include testosterone and androstenedione (A4) (89). To date, several articles have reported that WWE taking VPA have elevated androgen, hirsutism, or acanthosis (5, 14, 16, 72, 73, 76, 90).

**Figure 2:**
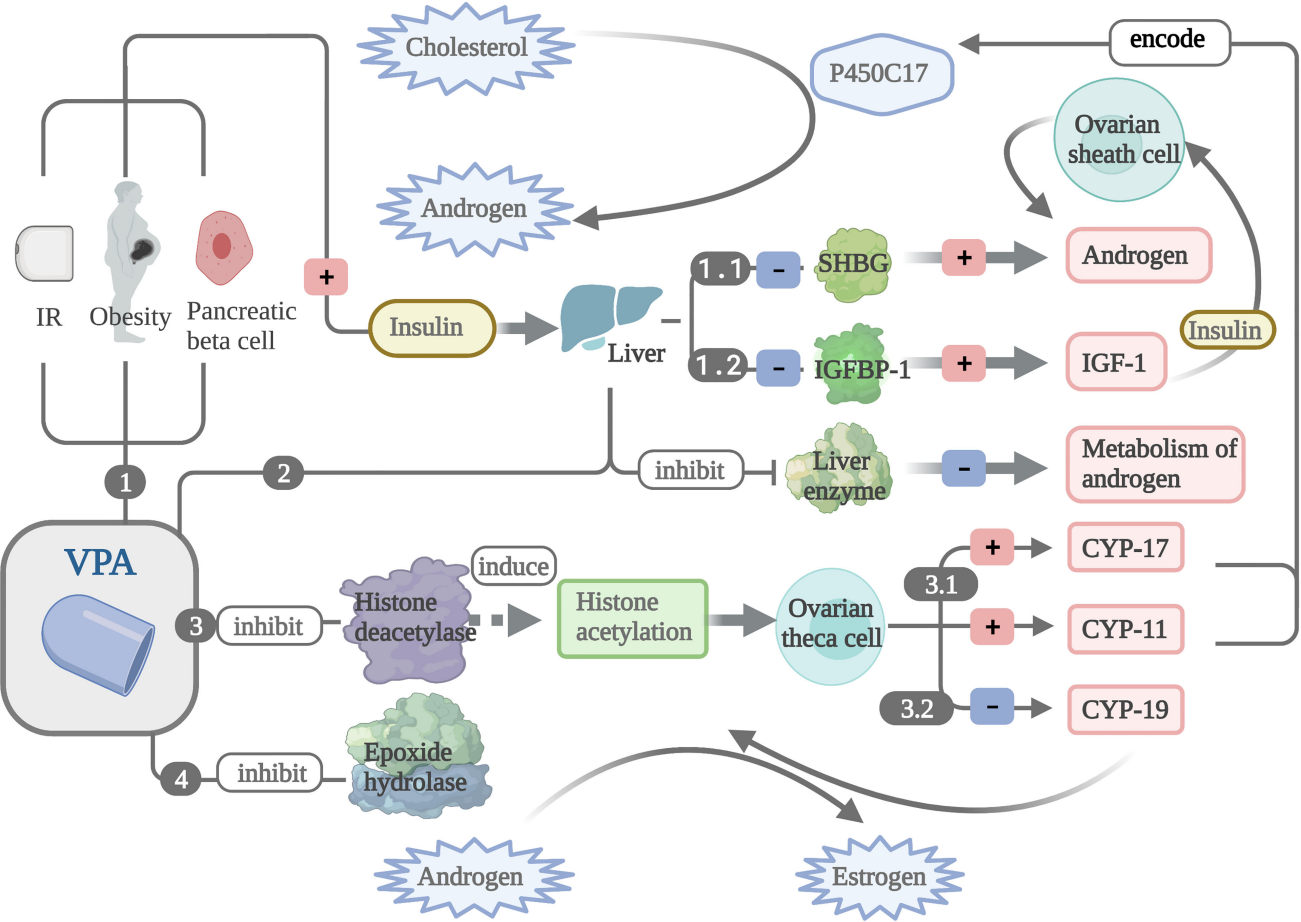
The mechanism of valproate (VPA) leading to increased level of androgen. *1.1* VPA can lead to hyperinsulinemia by inducing insulin resistance (IR) and weight gain and directly acting on pancreatic islet B cells, and then excessive insulin could hinder the synthesis of sex hormone binding globulin (SHBG) to elevate the level of androgen. *1.2* Insulin inhibits the production of insulin-like growth factor 1 binding protein (IGFBP-1) and increases the availability of (IGF-1). Insulin and IGF-1 increase the synthesis of androgen. *2* VPA can reduce the metabolism of androgens in the liver and increase androgen levels. *3.1* VPA potentiates androgen biosynthesis by promoting *CYP11* and *CYP17* gene expressions, encoding the P450 enzyme that participates in the conversion of cholesterol to androgen. *3.2* VPA also inhibits the expression of the *CYP19* gene encoding P450 aromatase to reduce the conversion of androgens to estradiol. *4* VPA can inhibit the activity of epoxidation hydrolase, which may also be involved in the conversion of androgens to estrogens.

The suggested mechanisms of the VPA-induced androgen level elevation include the following: 1) insulin resistance and weight gain are commonly associated with VPA treatment (6, 16, 81, 82). VPA can also directly impact pancreatic islet B cells to promote the secretion of insulin (91). The combined action of the three causes hyperinsulinemia, and then excessive insulin acts on the liver to hinder the synthesis of SHBGs (92, 93), which can increase the free testosterone level and inhibit the production of insulin-like growth factor 1 binding protein (IGFBP-1) (92). It was found to increase the availability of insulin-like growth factor 1 (IGF-1). Insulin and IGF-1 are the main extraovarian factors that regulate the synthesis of steroids (89), which can act on ovarian sheath cells to increase androgen synthesis, mainly enhancing LH-induced androgen secretion while having a less obvious effect on basic androgen production (94, 95). 2) VPA is a liver enzyme inhibitor that can reduce the metabolism of androgens in the liver and increase the androgen levels (76, 96) 3). VPA is a histone deacetylase inhibitor that affects chromatin modification by inducing histone acetylation (97, 98) and then potentiates androgen biosynthesis by promoting *CYP11* and *CYP17* gene expressions, encoding the P450 enzyme that participates in the conversion of cholesterol to androgen in human ovarian theca cells (99). VPA also inhibits the expression of the *CYP19* gene encoding P450 aromatase in human follicular cells. Therefore, VPA reduces the conversion of androgens to oestradiol, but its inhibition of *CYP19* only occurs in FSH-stimulated cells and cells with higher concentrations (100, 101). 4) Studies have found that the plasma levels of carbamazepine epoxide were higher when used in combination with VPA than when used alone, suggesting that VPA can inhibit the activity of epoxidation hydrolase (102, 103), which may also be involved in the conversion of androgens to oestrogens (104, 105). 5) The combined action of insulin and LH will significantly increase the expression of the *CYP17* gene in human ovarian theca cells to promote the synthesis of androstenedione (95, 106). 6) Some studies have shown that the production of steroids could be altered by VPA in adrenal cells due to its effect on cholesterol in the mitochondrial intima (107), so the increased androgen levels in patients may be partly from the adrenal gland (75). In addition, in mammals, insulin may enhance the frequency and amplitude of GnRH and LH pulsatile release by upregulating the expression of the *GnRH* gene (108) (**Figure 2**).

#### 4.1.3 The Possible Mechanism of Ovulation Disorder and Polycystic Ovary Induced by VPA

Several studies have also verified that PCO formation, increased LH levels, or increased LH/FSH ratio and menstrual disorders are commonly seen in patients treated with VPA (12, 16, 72, 73). Furthermore, an animal experiment showed that, compared with those of the control group, the quantity of follicles was significantly reduced, the atretic follicles were increased, and the ovary also showed multiple cystic follicles in the VPA group (109). The reported rate of polycystic ovaries in WWE is as high as 40% (110). The LH peak is necessary for ovulation. FSH promotes the development and maturation of follicles. When FSH decreases, it will affect the production, development, and maturation of follicles, leading to obstacles in follicular maturation, and no dominant follicle is selected. The LH level is normal or increased, but the LH peak cannot be formed, leading to failure of ovulation (40). A large number of follicles are atresic, and immature follicles that lack an antrum exist in the ovaries in the form of cysts (40, 46). The high androgen level in patients also affects the development and maturation of follicles (111). In addition, after treatment with VPA, the secretion of TGF-β1 in the follicle decreases (109), and the TGF-β superfamily exerts an indispensable effect in regulating the formation and development of follicles (112). In addition, some scholars proposed that VPA can participate in the process of apoptosis of ovarian cells by enhancing the level of the apoptotic hormone testosterone and activating the caspase-3-dependent apoptosis signaling pathway (100).

Menstrual disorders are a clinical manifestation of ODs and abnormal levels of sex hormones. A large number of reports have reported an increase in the incidence of menstrual disorders in patients with epilepsy or VPA treatment. The incidence of epilepsy in women is about 20%–35%, and even as high as 48% (39, 113–115). It can manifest as irregular menstrual cycles, oligomenorrhea, polymenorrhea, and even amenorrhea (5, 15, 39). Menstrual disorders are related to the age of onset (39), and they are more common in obese patients or those with IR (5). Moreover, the development of abnormal menstruation is not significantly related to the type of seizures, the duration of continuous use of VPA, and the dose of VPA (14, 116).

#### 4.1.4 The Possible Mechanism of IR/Hyperinsulinemia Caused by VPA

Under normal circumstances, insulin acts on the liver, fat cells, and skeletal muscles to maintain glucose homeostasis. The concept of IR refers to the reduction of insulin sensitivity due to various reasons, which can hinder the uptake and utilization of glucose; the body compensatively secretes too much insulin to maintain stable blood glucose levels. Among women with PCOS, the prevalence of IR is 44%–85% (117). Leptin, a hormone mainly derived from adipose tissue, participates in the regulation process of glucose, adipose, and energy metabolism, so it is able to indirectly regulate insulin sensitivity by reducing food intake and increasing energy consumption (79, 118). Adiponectin is a protein derived from fat cells that has an important regulatory effect on the insulin concentrations and glucose balance, regulating insulin sensitivity through a variety of mechanisms (79). Insulin resistance or increased insulin levels (5, 80–82, 119), increased leptin levels (79, 80), and decreased adiponectin levels (79, 82, 119, 120) have been reported in VPA subjects in a number of studies.

**Figure 3:**
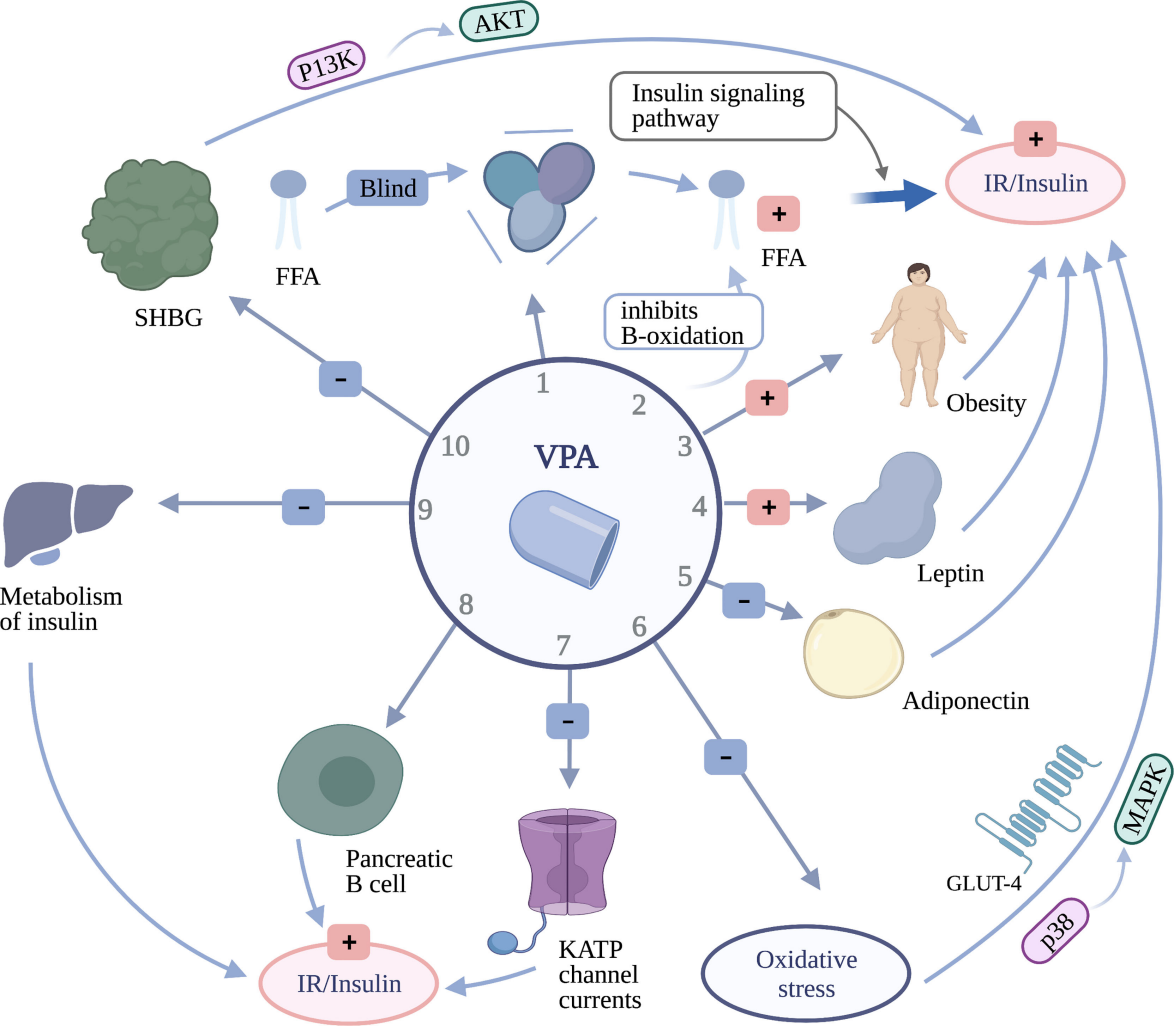
The mechanism of insulin resistance or hyperinsulinemia caused by valproate (VPA). *1* VPA can compete with free fatty acids (FFAs) for binding to albumin to increase the availability of FFAs, which can induce insulin resistance (IR) through the insulin signaling pathway. *2* VPA elevates the level of androgen by inhibiting the B-oxidation of FFAs. *3* Obesity promotes the occurrence of IR. *4* High leptin levels are closely related to IR. *5* The reduction of adiponectin is significantly related to IR. *6* VPA hinders oxidative stress, which can influence IR through the p38 MAPK signaling pathway and reduce the expression of glucose transporter 4 (GLUT-4). *7* VPA can attenuate K-ATP channel currents, which can then regulate the membrane potential of B cells, leading to increased insulin secretion. *8* VPA can directly act on pancreatic B cells to increase insulin secretion. *9* VPA could affect the metabolism of insulin in the liver, which can bring about an increase in insulin concentrations. *10* Refers to the level of sex hormone binding globulin (SHBG), which has an impact on the level of IR by regulating the PI3K/AKT signaling pathway.

The mechanism of VPA-induced IR or hyperinsulinemia has yet to be confirmed, but the following are potential mechanisms: 1) VPA is a short-chain and branched-chain fatty acid that can compete with free fatty acids (FFAs) for binding to albumin, increasing the availability of FFAs (121), and FFAs can induce IR *via* the insulin signaling pathway (82, 122). 2) VPA inhibits the B-oxidation of FFAs (123), which may be related to carnitine deficiency (124). The amount of FFAs affects insulin and glycemic responses (125). 3) VPA is related to obesity, as there is a close connection between obesity and IR, and obese patients have higher IR levels (119, 126). This effect may be involved in the high FFA levels and adipocytokines (127). 4) Due to its involvement in leptin and adiponectin signaling, the reduction of adiponectin is significantly related to IR (119, 128, 129), which can enhance insulin sensitivity by increasing fatty acid oxidation and inhibiting liver glucose production (82). High leptin levels are closely related to IR (80, 129). 5) Some researchers believe that VPA will injure the liver and affect the metabolism of insulin in the liver, which can bring about an increase in insulin concentrations (81, 130). 6) There are also studies that have found that long-term use of VPA can increase the levels of oxidative stress markers, such as malondialdehyde and myeloperoxidase (MPO) (79, 81, 91), and oxidative stress influences IR through insulin receptor signaling pathways, such as the p38 MAPK (mitogen-activated protein kinase) signaling pathway, and eventually reduces the expression of glucose transporter 4 (GLUT-4) (127, 131). In addition, excessive androgen was positively correlated with IR in PCOS patients (132), and exposure to VPA can attenuate ATP-sensitive potassium (K-ATP) channel currents, which can then regulate the membrane potential of B cells, leading to increased insulin secretion (133). 7) VPA can directly act on pancreatic B cells to increase insulin secretion (91). 8) Some researchers believe that VPA will injure the liver and affect the metabolism of insulin in the liver, which can bring about an increase in insulin concentrations (81, 130). 9) It may be related to SHBGs, but this needs to be verified, and it has an impact on the level of IR by regulating the PI3K/AKT signaling pathway. Its reduction contributes to the development of IR (93) (**Figure 3**).

### 4.2 Do Other ASMs Cause PCOS Through the HPO Axis?

#### 4.2.1 Levetiracetam

Research has shown that PCOS, oligomenorrhea, and excessive androgen increased in patients taking levetiracetam (LEV), and the proportions are 44%, 20%, and 24% respectively (134). LEV may also affect the HPG axis of female rats (135). This is consistent with the findings in male patients showing that LEV monotherapy may lead to changes in reproductive indicators through the hypothalamic–pituitary–testicular system (136). At present, the exact mechanism of the anti-epileptic effect of LEV is still unclear. It can bind to the synaptic vesicle protein SV2A in the brain and affect the SV2A–GABAergic system (137, 138). Therefore, LEV can also affect the HPO axis through GABA.

#### 4.2.2 Carbamazepine and Oxcarbazepine

Carbamazepine (CBZ) and oxcarbazepine (OXC) are the first-line or second-line alternatives for focal epilepsy and primary generalized tonic–clonic seizures (139). Patients with long-term CBZ treatment may show decreased levels of E2 and dehydroepiandrosterone sulfate (DHEA-S), increased levels of SHBGs, and menstrual disorders (140, 141). Part of the reason for these abnormalities may be the direct inhibition of the function of the hypothalamic–pituitary axis (141). The incidence of PCO in women treated with OXC is as high as 60%, and there will also be abnormal levels of dehydroepiandrosterone, testosterone, and SHBGs (142, 143). OXC can stimulate the GnRH neurons to release GnRH, thereby promoting the pituitary gland and testicles to secrete and release large amounts of FSH, LH, and testosterone (144).

#### 4.2.3 Phenytoin

Studies have shown that phenytoin (PHT) treatment adversely affected the HPG axis, induced the limbic system neurons to undergo apoptosis (145), increased the GABA levels, and induced the proliferation of GABA receptors. Therefore, PHT may affect the HPO axis by destroying neurons in the limbic system and GABA.

#### 4.2.4 Topiramate and Gabapentin

Topiramate (TPM) and gabapentin (CAS) treatment can interfere with sex hormone levels and also affect the GABAergic system and GnRH neuronal–glia plasticity (146). Therefore, TPM and gabapentin could destroy the completion of the HPG axis and cause reproductive dysfunctions through GABA or directly affect GnRH neurons.

#### 4.2.5 Lamotrigine

There is almost no adverse effect on female reproductive function, and it can even reverse the abnormal reproductive endocrine function caused by VPA, so lamotrigine (LTG) could be used as an alternative to VPA treatment (5, 147).

In short, after reviewing a large number of previous studies, it was found that there are only a few studies on the effects of traditional anti-seizure medications except VPA on the reproductive endocrine system of female patients, and the research on new anti-seizure medications lags behind. Their effect on reproductive endocrine is partly attributed to the liver enzyme-inducing properties of ASMs. For example, drugs with liver enzyme induction include PHT and CBZ, which can increase the levels of SHBGs and decrease the levels of testosterone (148). As a result, the HPO axis is affected by negative feedback, GABA, or other mechanisms. However, whether most drugs affect reproductive endocrine function through the HPO axis and how they affect the HPO axis remain to be further studied and determined (**Table 1**).

**Table 1:** The influence of anti-seizure medications (ASMs) on the hypothalamic–pituitary–ovarian (HPO) axis (5, 14, 16, 72, 134, 140, 142, 143).

ASMs	GnRH	LH	A	E2	SHBG	Related mechanism
VPA	NA	↑	↑	↓	NA	GABA levels, leptin and adiponectin levels, insulin levels, and protein modifications
LEV	NA	NA	↑	NA	NA	GABA negative feedback
CBZ	NA	NA	↓	NA	↑	Liver enzymes→SHBG androgen→negative feedback pathway
OXC	NA	NA	↓	NA	NA	GnRH neuron
PHT	↓	↓	↓	NA	↑	Liver enzymes→SHBG androgen→negative feedback pathway (limbic system neuron apoptosis/GABA)

ASMs, anti-seizure medications; GnRH, gonadotropin-releasing hormone; LH, luteinizing hormone; SHBG, sex hormone binding globulin; VPA, valproate; LEV, levetiracetam; CBZ, carbamazepine; OXC, oxcarbazepine; PHT, phenytoin; NA, not available.

## 5 Conclusion

PCOS-like reproductive endocrine disorder is a common complication in patients with epilepsy. Part of the reason is that the limbic system, a site closely related to epilepsy, has extensive and direct contact with the hypothalamus, so abnormal discharges can cause reproductive endocrine disorders through the HPO axis. Another reason is that, in view of the complex connections between neurotransmitters and epilepsy and GnRH neurons, abnormal levels of neurotransmitters may also cause reproductive endocrine disorders through the HPO axis. Consequently, VPA regulates the function of the HPO axis by affecting the GABA levels, leptin and adiponectin levels, insulin levels, and protein modifications. It can also affect reproductive endocrine metabolism by regulating signal pathways, affecting hormone metabolism, and other factors. Besides, some ASMs may affect the HPO axis through the negative feedback mechanism of sex hormones and GABA. Traditional anti-seizure drugs seem to have varying degrees of influence on the HPO axis. ASMs may be safer than traditional drugs, and their reproductive endocrine effects have not been extensively studied. In addition, the effects of epilepsy and anti-epileptic drugs on reproductive endocrine function vary with factors such as the type of epilepsy, the age at onset of seizures, the age when treatment was initiated, and the types of ASMs used. Patients with epilepsy are prone to reproductive endocrine disorders, especially obese patients or patients using VPA. Therefore, BMI, menstrual cycle, and sex hormone level changes should be checked regularly. In addition, those under the age of 20 years who start medication are also at high risk of reproductive endocrine disorders, especially female patients. Therefore, this part of the population must use medication carefully and must be closely monitored for reproductive function; new anti-seizure medications can be used as an alternative treatment, if necessary.

## Author Contributions

SL, LZ, NW, and ZT designed and wrote this article. ZX and YC helped with proofreading and revision. All authors contributed to the article and approved the final version.

## Conflict of Interest

The authors declare that the research was conducted in the absence of any commercial or financial relationships that could be construed as a potential conflict of interest.

## Publisher’s Note

All claims expressed in this article are solely those of the authors and do not necessarily represent those of their affiliated organizations, or those of the publisher, the editors and the reviewers. Any product that may be evaluated in this article, or claim that may be made by its manufacturer, is not guaranteed or endorsed by the publisher.
